# *slim shady* is a novel allele of *PHYTOCHROME B* present in the T‐DNA line SALK_015201

**DOI:** 10.1002/pld3.326

**Published:** 2021-06-12

**Authors:** Linkan Dash, Robert E. McEwan, Christian Montes, Ludvin Mejia, Justin W. Walley, Brian P. Dilkes, Dior R. Kelley

**Affiliations:** ^1^ Department of Genetics Development and Cell Biology Iowa State University Ames IA USA; ^2^ Center for Plant Biology Purdue University West Lafayett IN USA; ^3^ Department of Horticulture and Landscape Architecture Purdue University West Lafayett IN USA; ^4^ Department of Plant Pathology and Microbiology Iowa State University Ames IA USA; ^5^ Department of Biochemistry Purdue University West Lafayett IN USA

**Keywords:** auxin, cell elongation, hypocotyl, light signaling, phytochrome, T‐DNA

## Abstract

Auxin is a hormone that is required for hypocotyl elongation during seedling development. In response to auxin, rapid changes in transcript and protein abundance occur in hypocotyls, and some auxin responsive gene expression is linked to hypocotyl growth. To functionally validate proteomic studies, a reverse genetics screen was performed on mutants in auxin‐regulated proteins to identify novel regulators of plant growth. This uncovered a long hypocotyl mutant, which we called *slim shady*, in an annotated insertion line in *IMMUNOREGULATORY RNA‐BINDING PROTEIN* (*IRR*). Overexpression of the *IRR* gene failed to rescue the *slim shady* phenotype and characterization of a second T‐DNA allele of IRR found that it had a wild‐type (WT) hypocotyl length. The *slim shady* mutant has an elevated expression of numerous genes associated with the brassinosteroid‐auxin‐phytochrome (BAP) regulatory module compared to WT, including transcription factors that regulate brassinosteroid, auxin, and phytochrome pathways. Additionally, *slim shady* seedlings fail to exhibit a strong transcriptional response to auxin. Using whole genome sequence data and genetic complementation analysis with SALK_015201C, we determined that a novel single nucleotide polymorphism in *PHYTOCHROME B* was responsible for the *slim shady* phenotype. This is predicted to induce a frameshift and premature stop codon at leucine 1125, within the histidine kinase‐related domain of the carboxy terminus of PHYB, which is required for phytochrome signaling and function. Genetic complementation analyses with *phyb‐9* confirmed that *slim shady* is a mutant allele of *PHYB*. This study advances our understanding of the molecular mechanisms in seedling development, by furthering our understanding of how light signaling is linked to auxin‐dependent cell elongation. Furthermore, this study highlights the importance of confirming the genetic identity of research material before attributing phenotypes to known mutations sourced from T‐DNA stocks.

## 1 INTRODUCTION

In plants, phytohormones work in concert to coordinate cell divisions, cell expansions, and drive morphogenesis. Hypocotyl development in Arabidopsis has been an effective model system for studying the interaction between phytohormones and their roles in responses to various environmental signals. Arabidopsis hypocotyl has approximately 20 cell files from apex to base that do not proliferate post embryonically and their elongation largely depends on cell expansion (Gendreau et al., 1997). Consequently, post‐embryonic cell expansion determines hypocotyl elongation in Arabidopsis and is largely influenced by internal factors like hormones and external environmental factors like light and temperature.

Phytochromes are light sensing proteins that dynamically exist in two interconvertible isoforms, the red light‐absorbing Pr form and the far‐red light‐absorbing Pfr form that is considered to be biologically active (Rockwell et al., 2006). In light conditions, cytosolic biologically inactive Pr form absorbs red light and undergoes rapid conformational change to the Pfr form and is imported to the nucleus (Nagatani, 2004). Nuclear localized Pfr dimers physically interact with PHYTOCHROME INTERACTING FACTORS (PIFs), a group of basic helix‐loop‐helix transcription factors that bind to “G‐box” elements in promoters to facilitate transcription (Leivar & Monte, 2014; Leivar et al., 2012). Light‐activated phytochromes phosphorylate the PIFs and trigger their 26S proteasome‐mediated degradation and removing them from their target promoters (Al‐Sady et al., 2006; Park et al., 2012). The PIFs can directly trigger expression of downstream genes that can increase auxin biosynthesis and/or signaling (Franklin et al., 2011; Goyal et al., 2016; Hornitschek et al., 2012; Li, Ljung, et al., 2012; Nozue et al., 2011; Sun et al., 2012). The PIFs also regulate both brassinosteroid and auxin responses by physically interacting with their respective regulatory transcription factors, BRASSINAZOLE RESISTANT1 (BZR1), and AUXIN RESPONSE FACTORs (ARFs) (Oh et al., 2014).

The central signaling network that positively regulates hypocotyl growth in *Arabidopsis* consists of three major families of interdependently working transcription factors: BZR1, ARFs, and the PIFs, collectively referred to as the BZR/ARF/PIF module or the “BAP module” (Bouré et al., 2019). The BAP module works in concert to activate downstream genes that promote cell elongation in the hypocotyl (Favero, 2020). Plant hormone gibberellins also contribute towards hypocotyl elongation by modulating PIF activity and stability (Feng et al., 2008; Li et al., 2016; De Lucas et al., 2008). In addition, auxin is known to rapidly alter gene expression at the transcript and protein level in vegetative tissues including hypocotyls (Bargmann et al., 2014; Chapman et al., 2012; Clark et al., 2019; Nemhauser et al., 2006; Pu et al., 2019).

T‐DNA mutants are a valuable resource to the Arabidopsis community and have greatly facilitated genetic screens and functional genomics (Dilkes & Feldmann, 1998; O’Malley & Ecker, 2010; Page & Grossniklaus, 2002), but they are not without their drawbacks. Additional mutant alleles unassociated with T‐DNA insertions (also called untagged T‐DNA mutants) have been reported on numerous occasions and can be associated with duplications/translocations, insertion/deletions or other complex genetic changes (Tax & Vernon, 2001). Notable examples include second‐site mutations in the *abp1‐5* and *phyb‐9* backgrounds (Enders et al., 2015; Gao et al., 2015; Yoshida et al., 2018), a new set of *transparent testa* alleles (Jiang et al., 2020), and a mutation in *nrpd1a‐3* associated with ROOT HAIR DEFECTIVE 6 (RHD6) (David et al., 2019).

In this study, we performed a reverse genetic screen based on our auxin responsive proteomics data (Clark et al., 2019) and publicly available Arabidopsis T‐DNA insertion alleles (Alonso et al., 2003; O’Malley & Ecker, 2010). From this screen, we identified a T‐DNA line with a long hypocotyl phenotype which we initially called *slim shady* and was recently published as IRR (Dressano et al., 2020). A long hypocotyl phenotype in SALK_015201 lines had been previously reported (Petrov et al., 2013), but a second T‐DNA allele in *IRR* had a WT phenotype, prompting us to reconsider the genetic lesion responsible for the long hypocotyl phenotype. Analysis of whole genome sequence data available for this SALK line (Hu et al., 2019) identified a single base pair deletion within the coding sequence of *PHYB*. Transcriptomic comparison of SALK_015201 and WT seedlings with and without auxin determined that genes associated with the BAP regulatory module (Bouré et al., 2019) were altered in this T‐DNA background. Genetic complementation analyses confirmed the presence of a new *phyb* allele, which we call *slim shady*, in SALK_015201. This mutation is predicted to generate a truncated PHYB protein. This study serves as a reminder for the Arabidopsis community when working with T‐DNA alleles to backcross material, seek multiple alleles, and remain vigilant about the high rate of loss of function mutations in these lines.

## 2 MATERIALS AND METHODS

### 2.1 Plant material

All seed stocks used in this study were obtained from the Arabidopsis Biological Resource Center (ABRC) at Ohio State University. *Arabidopsis thaliana* plants used in this study were Columbia (Col‐0) ecotype. SALK_015201 encodes the *irr‐1* knock‐out allele (also annotated as SALK_015201C and SALKseq_015201) and SALK_066572 encodes the *irr‐2* knockout allele which were previously characterized (Dressano et al., 2020; Petrov et al., 2013). Homozygous *phyB‐9* seed stocks (ABRC stock number CS6217) were also previously characterized (Reed et al., 1994).

For transcriptomic profiling, Col‐0 and homozygous SALK_015201C seeds were surface sterilized using 50% bleach and 0.01% Triton X‐100 for 10 min and then washed five times with sterile water. Seeds were then imbibed in sterile water for two days at 4°C and then transferred to 0.5X Linsmaier–Skoog (LS) medium plates solidified with 0.8% agar and overlaid with sterile 100 micron nylon mesh squares to facilitate tissue harvesting. Seedlings were grown under long day photoperiods (16‐hr light/8‐hr dark) at 23°C. Six‐day‐old seedlings were treated with 1‐μM indole‐3‐acetic acid (IAA) dissolved in 95% ethanol (“auxin”) or an equivalent volume of 95% ethanol (“mock”) for 24 hr by transferring the seedlings on mesh squares to square petri dishes containing 10 ml of fresh 0.5X LS supplemented with IAA or mock solvent for the specified time. Following treatments, the seedlings were then harvested, weighed, and immediately frozen in liquid nitrogen; approximately 1 g of seedling tissue was collected per replicate/genotype. Four independent biological replicates were generated for each genotype and treatment.

For phenotyping assays, seeds were surface sterilized using 50% bleach and 0.01% Triton X‐100 for 10 min and then washed five times with sterile water. Seeds were then imbibed in sterile water for two days at 4°C and then transferred to 0.5X LS medium plates. Seedlings were grown under long day photoperiods (16‐hr light/8‐hr dark) and dark (24‐hr dark post 2‐hr light incubation) at 23°C. For auxin response assays, 5‐day‐old seedlings were transferred to either 0.5X LS plates or 0.5X LS plates supplemented with 1‐μM IAA and grown for another 2 days.

A *35S:GFP‐IRR* transgenic line was created using Agrobacterium mediated transformation into the SALK_015201C background. Seven‐day‐old Col‐0 seedlings were snap frozen in liquid nitrogen followed by tissue lysis using mortar and pestle. Extraction of total RNA from the harvested tissue was done using TRIzol™ Reagent (Invitrogen, catalog number‐15596026) followed by purification using RNeasy® mini‐kit (Qiagen, catalog number‐74106). The purified total RNA was used to synthesize cDNA using SuperScript™ IV Reverse Transcriptase kit from Invitrogen (catalog number‐18090010). The synthesized total cDNA was used as a template for PCR amplifying the full‐length *IRR* cDNA, using primers modified for directional cloning (Table S2). The blunt end PCR product was cloned into the pENTR™/D‐TOPO^®^ donor vector, using the gateway cloning kit provided by Invitrogen (catalog number: K240020). The entry clone was then transformed into chemically competent DH5α cells. Transformants were selected for kanamycin resistance and tested by colony PCR and restriction digest. The cloned plasmids were then isolated from their bacterial cultures using GeneJET Plasmid Miniprep Kit from Thermofisher scientific (catalog number‐K0503). In the next step, an LR reaction was set between the entry clone and the gateway destination vector pGWB606 (Nakamura et al., 2010) using LR clonase™ enzyme mix provided by Invitrogen (catalog number‐11791020). In this case, the transformants in *Escherichia coli* were selected by spectinomycin resistance, colony PCR with primers specific to the chimeric *GFP:IRR* cDNA (Table S2). The final clone was further confirmed by sanger sequencing (data not shown), followed by their transformation into chemically competent Agrobacterium cells of GV3101 strain. Columbia‐0 and SALK_015201 plants were grown under long day photo period (16‐hr light, 8‐hr dark) until they reached bolting. Floral dip plant transformation protocol was performed on these plants, and they were left to grow normally until senescence. Harvested seeds from the dipped plants were sown on soil and were allowed to grow normally until 2–4 leaves stage. Juvenile plants were sprayed with a 1:1,000 dilution of 120mg/ml BASTA (Finale) for selecting the positive transformants which were further confirmed through genotyping for the presence of chimeric *GFP‐IRR* gene (data not shown).

### 2.2 Genotyping

All primers used for genotyping are provided in Table S2. Primers to genotype SALK alleles were designed using the SALK T‐DNA verification primer design tool at the SALK SiGNAL website (http://signal.salk.edu/tdnaprimers.2.html). A derived cleaved amplified polymorphism (dCAPS) marker was designed to differentiate between the WT and *slim shady* alleles of *PHYB* using the dCAPS 2.0 finder tool (Bui & Liu, 2009). For the dCAPS analysis, a ~280‐bp region of *phyB* was amplified using the dCAPS primers (Table S2) using DreamTaq polymerase (Thermo Fisher Scientific) and subsequently digested with ApoI‐HF (New England Biolabs) according to manufacturer's protocols. The digested PCR products were analyzed on a 4% agarose gel. The WT allele of *PHYB* is expected to be cleaved by ApoI‐HF, while the mutant *slim shady* allele is not cleaved.

### 2.3 Genetic analyses

Homozygous single mutants were crossed to generate F1 seedlings for complementation analyses: SALK_015201C was crossed to both Col‐0 and *phyb‐9*. As a control, *irr‐2* was also crossed to *phyb‐9*. The resulting F1 seeds were surface sterilized as described above and plated on 0.5X LS for phenotyping.

### 2.4 Transcriptomic and GO enrichment analyses

RNA was extracted from 0.1 g of 7‐day‐old seedlings (Col‐0 and SALK_015201) using Trizol followed by column clean up using the Quick‐RNA plant kit (Zymo). Total RNA concentrations were determined using a NanoDrop and Qubit. RNA quality was checked via Bioanalyzer at the ISU DNA Facility. QuantSeq 3′ mRNA libraries were prepared using the Lexogen 3′ mRNA‐seq FWD kit and sequenced on an Illumina HiSeq 3000 as 50 bp reads at the ISU DNA facility. QuantSeq reads were mapped to the TAIR10 genome and differential gene expression analysis was performed using PoissonSeq implemented in R (Li et al., 2012). Transcripts with a FDR cutoff of <0.05 and a log fold change ≥1.75 were defined as high confidence differentially expressed. Gene Ontology (GO) enrichment analyses were performed using BiNGO in Cytoscape (Maere et al., 2005) with either upregulated or downregulated genes as the input.

### 2.5 SNPs calling and informatics

DNA polymorphisms were identified following alignment of whole‐genome sequences to the *A. thaliana* TAIR10 genome. Genomic sequence data for *A. thaliana* accessions SALK_015201 and CS85255 were retrieved from the Sequence Read Archive (SRA) at the National Center for Biotechnology Information (NCBI) with IDs SRR5249176 and SRR5249156, respectively (Hu et al., 2019). These paired‐end 150‐bp reads were mapped to the TAIR10 reference genome (Lamesch et al., 2012) using BWA‐MEM (version 0.7.17) (Li & Durbin, 2009). Duplicate reads were identified and removed from alignment files using the *rmdup* command of SAMtools (version 0.1.18) (Li & Durbin, 2009). SNP and small indel calling was carried out as a three‐step process. First, the SAMtools *mpileup* command was used to transpose mapped reads to the reference genome and compute genotype likelihood. Next, the BCFtools *view* command was used to perform actual variant calling, and finally, preliminary quality filtering was done with *varFilter* command of the *vcfutil.pl* script with the “‐D100” option to exclude variants with more than 100 reads coverage. Further filtering was performed with SnpSift (version 3.6) (Cingolani et al., 2012) to keep only variants that have a phred‐scale quality of at least 20. The VCFtools *exclude‐positions* command was used to remove false‐positive variants using a list of variants common to phenotypically unaffected individuals. These variants are deemed false positives because they occur in numerous lineages and thus cannot be causal for a phenotype seen only in a single lineage (Addo‐Quaye et al., 2017, 2018). SnpEff (version 3.6) (Cingolani et al., 2012) was used with TAIR10 gene annotation to predict the effect of the remaining variants on gene function.

To check for incomplete integration by the T‐DNA or Ti plasmids and confirm the identities of the SRA samples, sequences for pDs‐Lox, pBIN‐pROK2, and M11311.1 Ti plasmid were added as extra chromosomes to the TAIR10 reference genome to create a new TAIR10 pseudo‐reference genome that could detect these T‐DNA sequences by alignment. Reads from SRR5249176 and SRR5249156 were independently aligned to the pseudo‐reference with BWA‐MEM. Alignment files were converted from SAM to BAM format with the SAMtools *view* command prior to duplicate read removal with the *rmdup* command. Deduplicated BAM files for both samples were then viewed in IGV to check for the presence of Ds‐Lox and pBIN‐pROK2 T‐DNA.

### 2.6 RNA polymorphism calling by alignment to *A. thaliana*

RNA‐seq reads for 12 individuals comprising Pep‐treated and untreated *irr‐1* mutant and WT plants (three biological replicates per genotype per condition) were obtained from the NCBI SRA with IDs SRR11218900–SRR11218911 (Dressano et al., 2020). The paired‐end 150‐bp reads were aligned to the TAIR10 reference genome (Lamesch et al., 2012) using the spliced alignment option of the STAR aligner (Dobin et al., 2013). The output was processed for duplicate read removal with SAMtools *rmdup* and passed to the variant calling pipeline described above, with the notable exception of the exclusion of a false‐positive variant removal step.

Aligned reads were additionally analyzed to identify differentially expressed genes (DEGs) induced by Pep treatment using DESeq2 (Love et al., 2014). The *htseq‐count* function of HTSeq (Anders & Huber, 2010) was used to project alignment files to TAIR10.32 gene/transcript annotation to count reads/fragments to produce gene level count matrices used as input for DESeq2. Genes were considered differentially expressed between Pep treated versus untreated if they had an adjusted *p* value ≤.1 (i.e., false discovery rate of 0.1 was applied).

### 2.7 Hypocotyl and root phenotyping

Intact seedlings (7‐day‐old) were imaged on a flat‐bed scanner (Epson V600). Measurements of hypocotyl and primary root lengths were performed using ImageJ. One‐way analysis of variance (ANOVA) was performed for comparing the F1 and single mutant genotypes against control Col‐0. Here, the hypocotyl length was taken as the dependent variable with genotype of the plants as the only independent variable.

### 2.8 Sanger sequencing and sequence alignments

A 644‐bp region of *PHYB* was amplified from Col‐0, SALK_015201, and *irr‐2* genomic DNA using gene specific primers (Table S2) with Phusion polymerase (Thermo Fisher Scientific) and cleaned up using the GeneJET PCR Purification Kit from Thermofisher (catalog number K0701). Purified PCR products were subjected to standard Sanger sequencing at the Iowa State University DNA Facility on an Applied Biosystems 3730xl DNA Analyzer. Sequence files were trimmed in SnapGene and aligned using CLUSTAL Omega.

### 2.9 Accession numbers

Sequence data from this article can be found at the NCBI BioProject database under SubmissionID: SUB8925253 and BioProject ID: PRJNA694682. The project information will be accessible with the following link within a few days of the release date upon publication: http://www.ncbi.nlm.nih.gov/bioproject/694682. Data analyses performed herein utilized public repository data from Hu et al., (2019) *BMC Bioinformatics* deposited at the NCBI SRA database under accession SRR5249176 and (Dressano et al., 2020) deposited at NCBI GEO GSE146282.

## 3 RESULTS

### 3.1 SALK_015201 (*irr‐1*) displays a long hypocotyl phenotype

Auxin‐regulated gene expression has been well‐characterized and influences many aspects of seedling growth and development. We recently quantified auxin‐regulated proteome remodeling in 5‐day‐old Arabidopsis seedlings (Clark et al., 2019; Kelley et al., 2017) and found widespread changes in protein abundance not linked to transcriptional changes. In order to explore and test this idea further, we examined auxin responsive proteins from our dataset to identify putative candidate proteins which could underlie post‐transcriptional auxin‐mediated gene expression. One top candidate was the protein IMMUNOREGULATORY RNA‐BINDING PROTEIN (IRR; AT3G23900) which increased in abundance in response to auxin (Kelley et al., 2017) and is known to influence mRNA splicing in Arabidopsis (Dressano et al., 2020).

**FIGURE 1:**
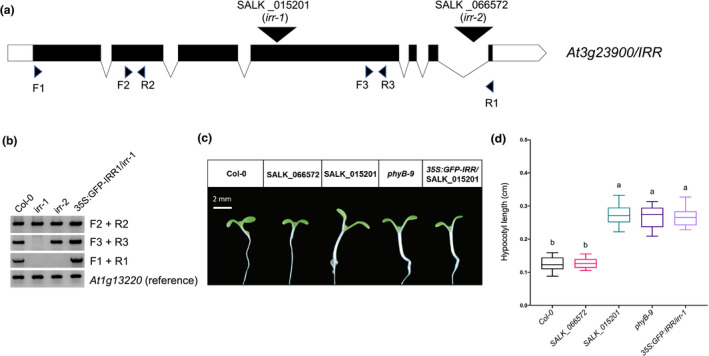
(a) Annotated T‐DNA alleles of *IMMUNOREGULATORY RNA BINDING PROTEIN* (*IRR*) include SALK_015201 (*irr‐1*) and SALK_066572 (*irr‐2*). Exons are indicated as black bars, introns as lines, untranslated regions (UTR) as white boxes and T‐DNA insertions as inverted triangles. (b) RT‐PCR of these alleles indicates that both *irr‐1* and *irr‐2* are null alleles of *IRR* that produce upstream truncated transcripts, while a *35S::GFP‐IRR* transgenic line in the SALK_015201 background has restored expression of *IRR*. *At1g13220* was used as a control gene. (c) Seven‐day‐old *phyb‐9,* SALK_015201 and *35S:GFP‐IRR/irr‐1* seedlings display long hypocotyls compared to Col‐0 and *irr‐2*. Scale bar = 2 mm. (d) Quantification of hypocotyl lengths. Letters a and b indicate the significant statistical differences between the hypocotyl lengths of different genotypes, determined by a one‐way ANOVA followed by Tukey's HSD post hoc test with *p* < .05

In order to characterize loss of function of *IRR* and determine if IRR is required for auxin regulated gene expression, we obtained two publicly available SALK T‐DNA lines in this gene (Figure 1). Initial phenotyping of the SALK_015201C line indicated that this T‐DNA line exhibited a long hypocotyl phenotype (Figure 1), which had been previously reported (Petrov et al., 2013). Conversely, the other SALK allele annotated in the *IRR* gene, SALK_066572 had a WT seedling phenotype. Based on our initial phenotypic characterization, we termed the SALK_015201C mutant *slim shady*. Notably, 15‐day‐old *irr‐1* (SALK_015201) and *irr‐2* (SALK_066572) roots have been described as longer compared to WT (Dressano et al., 2020), but we did not observe any differences in primary root length in either SALK allele compared to WT (data not shown). This could be due to different growth media conditions and/or developmental age examined between the studies. Homozygous seed stocks of SALK_015201C and SALK_066572 were confirmed using gene specific and T‐DNA primers listed in Table S2.

To characterize *IRR* gene expression in SALK_015201C (*irr‐1*) and SALK_066572 (*irr‐2*), we performed reverse transcription polymerase chain reaction (RT‐PCR) analysis using gene specific primers (Table S2) designed to amplify either full‐length transcript or truncated mRNA amplicons both upstream and downstream of the T‐DNA insertion sites (Figure 1a). *At1g13220* was used as a reference gene for normalization (Czechowski, 2005). Consistent with a previous report, neither allele produced a full‐length *IRR* transcript, nor truncated upstream transcripts were detected in both alleles (Figure 1b). *IRR* expression was examined using primers that amplified target regions in exon 2 (upstream of the annotated T‐DNA insertion site), exon 4 (downstream of the annotated T‐DNA insertion sites) as well as the full length of *IRR* cDNA. The expression of a *35S:GFP‐IRR* transgene in the SALK_015201 background restored expression of *IRR* back to normal (Figure 1b).

Loss of phytochrome function is classically associated with a long hypocotyl phenotype (Franklin & Quail, 2010). As a phenotypic comparison, we quantified the hypocotyl lengths of the SALK alleles and the *35S:GFP‐IRR*/SALK_015201 line relative to WT and *phyb‐9* (Figure 1c). The SALK_015201 line exhibits an elongated hypocotyl phenotype as compared to the Col‐0 and SALK_066572 (*irr‐2*) (Figure 1d). While the hypocotyl length of 7‐day‐old SALK_015201 seedlings was found to be statistically indifferent from *phyB‐9* (Figure 1d). Transgenic lines expressing *35S:GFP‐IRR* failed to restore the hypocotyl phenotype in SALK_015201 back to normal (Figure 1c,d).

### 3.2 BAP module gene expression is elevated in SALK_015201 seedlings

**FIGURE 2:**
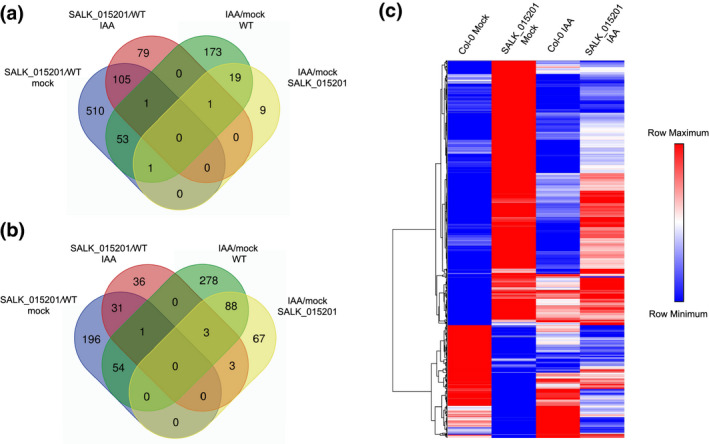
Differential gene expression (DEG) analysis in SALK_015201 compared to Col‐0. Venn diagrams of (a) upregulated genes and (b) downregulated genes. (c) Heat map of all 952 DEGs depicting their mean normalized read counts where the scale bar ranges from lowest (blue) to highest (red) relative expression value within a row generated by hierarchical clustering using one‐minus Pearson correlation matrix

**FIGURE 3:**
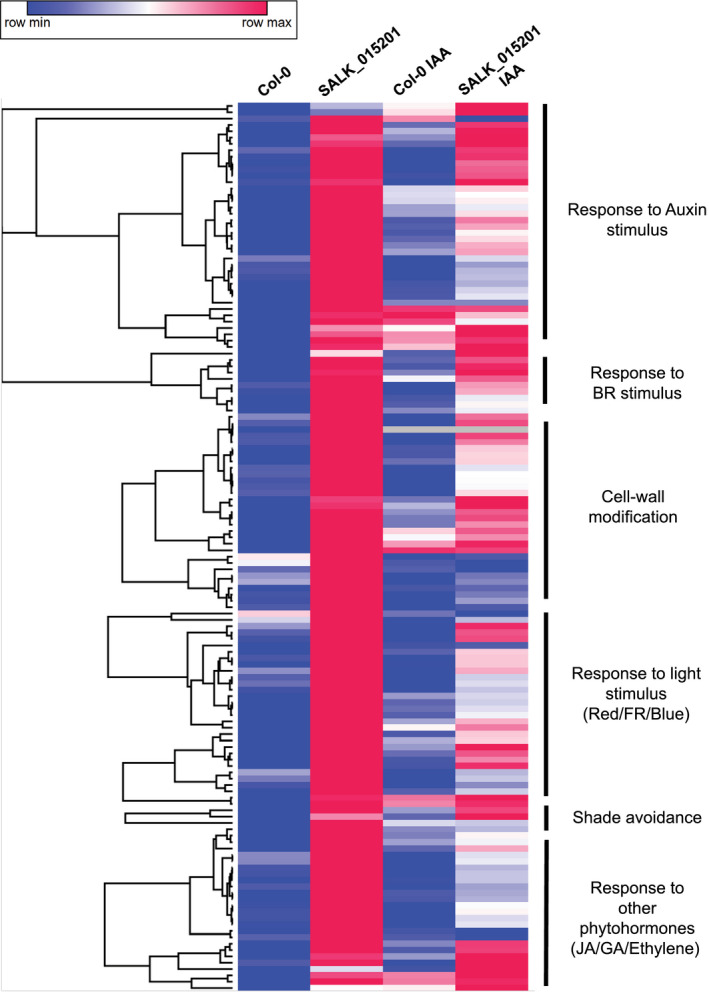
Heatmap of enriched genes in SALK_015201 compared to Col‐0 identified by GO analysis. The mean normalized read counts of selected genes with their GO term annotations are shown. The scale bar ranges from lowest (blue) to highest (magenta) relative expression value within a row

We hypothesized that the long hypocotyl phenotype observed in SALK_015201 seedlings may be due to altered auxin responses and/or light signaling. To test this hypothesis, we performed RT‐qPCR on three key marker genes, IAA INDUCIBLE 14/SOLITARY ROOT (*IAA14/SLR*), *PHYTOCHROME A* (*PHYA*), and *PHYB*. *PHYA* and *IAA14* were highly expressed in the mutant, whereas the *PHYB* transcript showed no significant change by RT‐qPCR (Figure S1). We then performed a transcriptomic analysis to determine global gene expression patterns in SALK_015201 both with and without auxin treatment using the 3′ end mRNA sequencing method (Moll et al., 2014). Differential gene expression (DE) analysis was determined using the PoissonSeq package implemented in R (Li, Witten, et al., 2012). We applied an FDR threshold of 0.05 and a log fold change between comparisons (i.e., WT versus SALK_015201 mock treated) ≥1.75 to define DE genes. We found 670 genes upregulated and 282 genes to be downregulated in SALK_015201 compared to WT (Figure 2 and Table S1). In response to a 24‐hr auxin treatment (1‐mM IAA) we observed 186 genes upregulated and 74 genes to be downregulated in SALK_015201 compared to WT (Figure 2 and Table S1). GO terms such as auxin response, light response (blue, red, and far‐red), cell wall modification, and shade avoidance response were enriched for the set of highly expressed genes in SALK_015201 as compared to the WT (Figure 3 and Figure S2). Downregulated genes were enriched in GO terms such as nuclear mRNA splicing via spliceosome, glucosinolate biosynthetic process, response to jasmonic acid stimulus (Figure S3).

A previous analysis of SALK_015201 included transcriptomic profiling of 15‐day‐old seedling tissue from this line (Dressano et al., 2020). A comparison to between this dataset and our transcriptomic analysis identified 101 common DEGs, with 85 common upregulated and three common downregulated genes. In addition, 13 of these genes were differentially expressed in the opposite direction (Table S3). Some of the notable GO terms that were enriched by these 101 common DEGs are “response to ethylene,” “response to oxidative stress,” and “response to chemical stress.” We also acknowledge that, because the QuantSeq and RT‐qPCR were performed on SALK_015201, which turns out to be a double mutant (*irr‐1 phyb*), the data presented here represent DE genes in this stock line and are not a consequence of one mutation alone per se.

We observed that multiple transcription factors belonging to the BAP module (i.e., BZR/ARF/PIF families) (Bouré et al., 2019) were upregulated in SALK_015201 as compared to the WT consistent with the elongated hypocotyl phenotype. Classic negative regulators of photomorphogenesis such as *BBX28*, bHLH transcription factors *PIF4*, *PIF3*, *PIL1*, and *PIL6* (Dong et al., 2017; Fujimori et al., 2004; Huq & Quail, 2002; Lin et al., 2018; Luo et al., 2014) showed an increased transcript abundance in SALK_015201 as compared to the WT (Figure 3 and Table S1). We also found two of the nine major photoreceptors upregulated in SALK_015201 including the UVA/Blue light‐sensing *PHOT1* (Briggs & Olney, 2001) and far‐red light sensing *PHYA* (Smith, 2000) (Figure 3 and Table S1). Our results are congruent with a previous report which discussed the importance of PHYB in photo‐regulation of *PHYA* gene expression in plants grown under constant white light or red light (Cantón & Quail, 1999).

Auxin plays a critical role in hypocotyl growth by driving cell expansion (Gray et al., 1998; Zhao et al., 2001). Consistent with this phenomenon, we observed numerous auxin inducible genes such as *IAA1*, *IAA2*, *IAA7*, *IAA29*, *ARF19*, and *ARGOS* to be highly expressed in SALK_015201 compared to WT. Also, genes involved in auxin homeostasis such as *GH3.3*, *WES1*, *DFL1*, and *NIT2* were upregulated in the mutant. Earlier studies have linked *SMALL AUXIN UP‐RNA* (*SAUR*) transcript abundance changes to hypocotyl elongation (Chae et al., 2012; McClure & Guilfoyle, 1987; Spartz et al., 2012; Stamm & Kumar, 2013). Overexpression of *SAUR15* and *SAUR36* promotes hypocotyl elongation because of enhanced cell expansion (Spartz et al., 2014; Stamm & Kumar, 2013). Additionally, *SAUR19*, *SAUR21*, and *SAUR24* are rapidly induced in the elongating hypocotyls of Arabidopsis seedlings grown in dark (Spartz et al., 2012). Consistently, we found 18 *SAUR* genes to be overexpressed in SALK_015201 compared to WT which is consistent with the observed phenotype (Table S1). ABCB19, a protein that modulates hypocotyl growth and is required for polar auxin transport (Wu et al., 2016), also showed transcript abundance in SALK_015201 (Table S1). Collectively, a large suite of auxin pathway genes were upregulated in SALK_015201 suggesting that altered auxin response and/or metabolism may contribute to the elongated hypocotyl phenotype.

Cell expansion plays a key role in plant growth and development (Braidwood et al., 2014) and serves as the fundamental process that drives hypocotyl elongation in etiolated *Arabidopsis* seedlings (Gendreau et al., 1997). EXTENSIN (EXT) and EXPANSIN (EXPs) are two major protein families with known roles in plant cell wall extension and modification (Cosgrove, 2000; Lamport, 1966; McQueen‐Mason et al., 1992). We found five *EXP*, four *EXT*, and five *XTH* mRNAs to be elevated in SALK_015201 that might explain its elongated phenotype.

The importance of gibberellic acid (GA) as a key phytohormone in regulating the shoot development was determined through genetic and biochemical studies (Jacobsen et al., 1996; Jacobsen & Olszewski, 1993; Koorneef et al., 1985; Peng et al., 1997). Additionally, exogenous application of bioactive GA is known to promote hypocotyl growth in light grown seedlings (Cowling & Harberd, 1999). Congruently, we observed an elevated gibberellin dependent gene expression response in SALK_015201 as compared to the WT (Table S1). Genes such as *GA‐STIMULATED ARABIDOPSIS 6* (*GASA6*) and *GASA14* were highly expressed in the mutant, indicating an altered GA activity in the mutant.

### 3.3 Identification of a background *phyB* mutation, *slim shady*, in SALK_015201

Expression of a *35S:GFP‐IRR* transgene in the SALK_015201 background failed to complement the long hypocotyl phenotype (Figure 1c,d), leading us to suspect that SALK_015201 may harbor an unlinked second‐site mutation in addition to the annotated T‐DNA insertion in *IRR*. Such mutations have been observed in numerous other T‐DNA lines (David et al., 2019; Enders et al., 2015; Gao et al., 2015; Jiang et al., 2020; Yoshida et al., 2018) and are thought to be an unintended consequence of the T‐DNA integration process (Castle et al., 1993; Laufs et al., 1999; Nacry et al., 1998; Negruk et al., 1996; Tax & Vernon, 2001).

**FIGURE 4:**
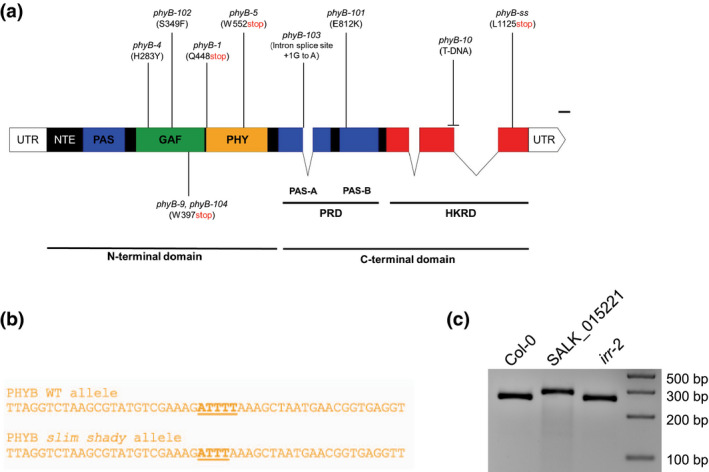
(a) Whole genome sequencing of SALK_015201 indicates a single base pair deletion in *PHYTOCHROME B* at position 3,370 which would lead to a premature stop codon after amino acid L1125 within the histidine kinase‐related (HKR) domain. (b) A derived cleaved amplified polymorphic sequence (dCAPS) marker was designed to differentiate between the WT and *phyb‐ss* alleles of *PHYB*. (c) Genotyping Col‐0, SALK_015201 and *irr‐2* lines for *PHYB* and *phyb‐ss* alleles using the dCAPS marker. Both Col‐0 and *irr‐2* are homozygous for the WT *PHYB* allele while SALK_015201 is homozygous for the *phyb‐ss* allele

Previously, a whole genome sequence was performed for SALK_015201 (Hu et al., 2019). Remapping and variant call analysis of these genomic sequences identified a single base pair deletion of a T nucleotide in the *PHYTOCHROME B* gene (AT2G18790) at position Chr2:8,144,002, which corresponds to position 3,370 in the *PHYB* cDNA (Figure 4a and Figure S7). We confirmed the single nucleotide deletion through a dCAPS assay (Neff et al., 1998) where we introduced an ApoI restriction enzyme site by using primers that contained a single nucleotide mismatch to the template DNA. In this assay, the WT *PHYB* allele gets digested into two fragments of 24 and 254 base pairs while the mutant *slim shady* allele cannot be cleaved. The SALK_015201 genomic DNA produced a single 277‐bp band in this assay while Col‐0 and *irr‐2* produced the expected WT *PHYB* allele PCR products (Figure 4c).

We also performed PCR and Sanger sequencing reactions on genomic DNA samples from Col‐0, SALK_015201, and *irr‐2* to confirm this single thymine deletion within the *PHYB* locus. This analysis confirmed the presence of a single base pair deletion in *PHYB* in the SALK_015201 background that was not observed in Col‐0 or *irr‐2* (Figure S4).

This single nucleotide deletion in the *PHYB* coding sequence is predicted to result in a conversion of L1125 to a stop codon in the C‐terminal region of the PHYB protein and lead to a truncated protein lacking the last 48 amino acids after I1124 (Figure 4a). The annotated histidine kinase domain of PHYB occurs at amino acid positions 934–1153; thus, the *slim shady* allele of the PHYB would lack a functional kinase domain. The predicted truncated protein would be similar to previously reported C‐terminal domain mutations of phytochrome B which reduce its biological activity (Matsushita et al., 2003; Qiu et al., 2019; Wagner et al., 1996; Wagner & Quail, 1995).

### 3.4 Genetic complementation analyses confirm that *slim shady* is an allele of *phyB*

**FIGURE 5:**
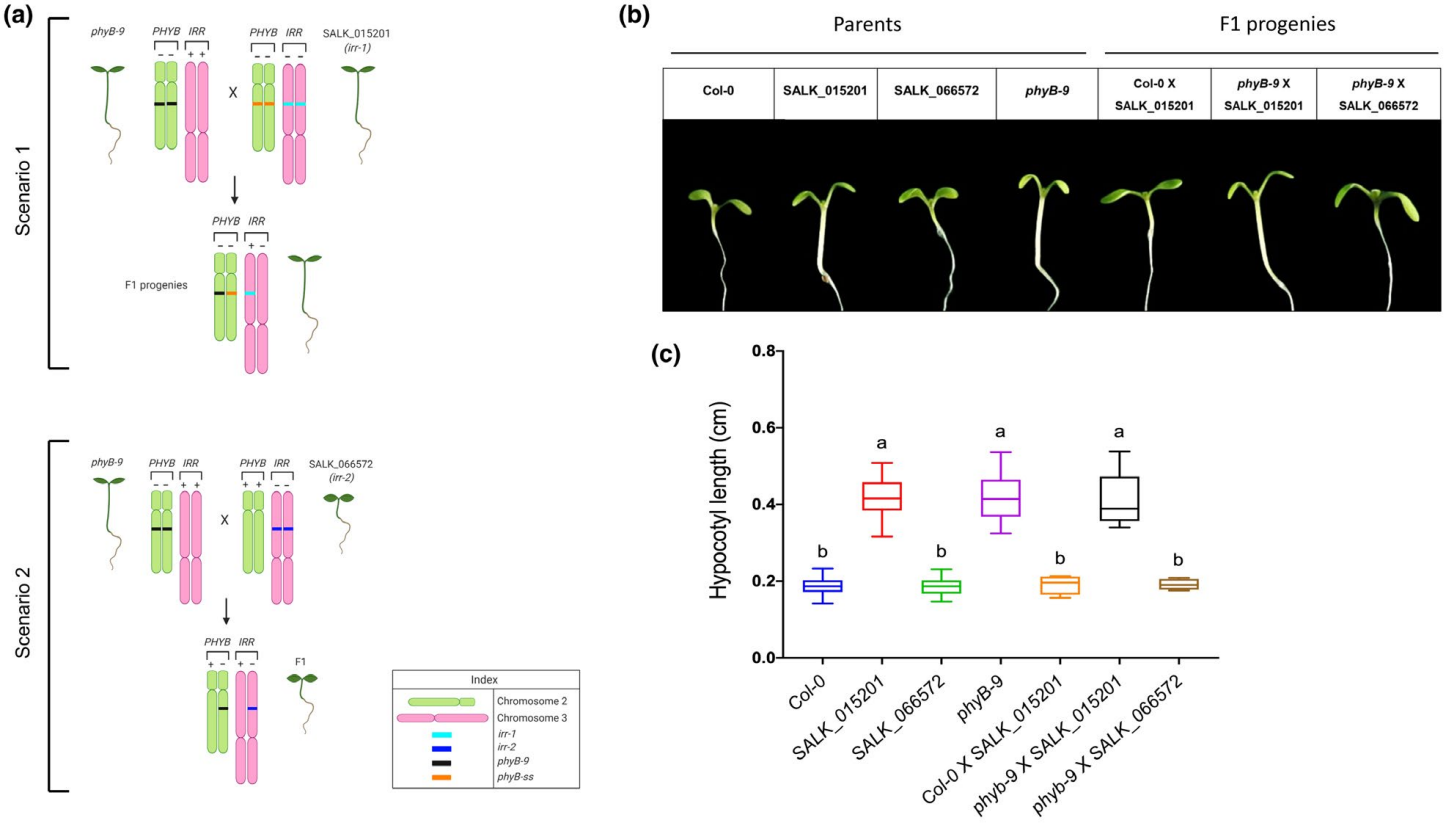
(a) Cartoon schematic of genetic complementation analyses. In scenario 1, the genetic complementation fails because the *phyB‐ss* mutation in SALK_015201 and the resulting F1 offspring have long hypocotyls. In scenario 2, the genetic complementation occurs because the SALK_066572 (*irr‐2*) background contains a wild‐type *PHYB* allele and the resulting F1 offspring have a wild‐type hypocotyl length. (b) Light micrographs of parental and F1 offspring seedlings showing representative hypocotyl lengths. Scale bar = 2 mm. (c) Quantification of hypocotyl lengths following genetic complementation analyses. Letters “a” and “b” indicate the significant statistical differences between the hypocotyl lengths (cm) of different genotypes, determined by a one‐way ANOVA followed by Tukey's HSD post hoc test with *p* < .05. Each color represents a unique genotype

The long hypocotyl phenotype we observed in SALK_015201 is due to a loss‐of‐function allele in *PHYB*. We predicted that *phyb‐9* would fail to complement the long hypocotyl phenotype in SALK_015201 (Figure 5a). Conversely, *irr‐2* should complement *phyb‐9*. We crossed *phyB‐9* with both SALK_015201 and *irr‐2* and collected F1 seed. F1 seeds and homozygous parental lines were plated on 0.5X LS media supplemented with 1% sucrose to examine hypocotyl phenotypes. The *phyB‐9 x irr‐2* F1 plants displayed wildtype hypocotyl phenotypes, but all F1 offspring from the *phyB‐9 x* SALK_015201 crosses exhibited long hypocotyl phenotype (Figure 5b). Thus, *slim shady* represents a novel loss of function allele in *PHYB*.

## 4 DISCUSSION

We recently characterized auxin responsive proteomes (Kelley et al., 2017) and used these data to perform a reverse genetic screen (Page & Grossniklaus, 2002) for seedlings with altered auxin‐dependent phenotypes. One of the candidate genes included in this screen was AT3G23900 which has been recently characterized as *IRR*, a novel RNA‐binding protein that is involved in alternatively splicing of its target genes that serve as key defense regulators against pathogen in Arabidopsis (Dressano et al., 2020). Our initial screen was performed with hundreds of SALK T‐DNA insertion lines obtained as homozygous so‐called SALK “C” lines from the ABRC (O’Malley & Ecker, 2010) including SALK_015201C which contains a knock‐out allele of *IRR*, designated as *irr‐1* in (Dressano et al., 2020). This allele shows an elongated hypocotyl phenotype (Figure 1) which has been reported earlier (Petrov et al., 2013). Expressing a *35S:GFP‐IRR* in the SALK_015201C background failed to rescue the WT hypocotyl phenotype (Figure 4) that leads us to suspect the presence of a second‐site mutation in this SALK line.

A single base pair deletion in the *phyB* locus within the last exon of the coding sequence was identified through remapping and variant call analyses of publicly available whole genome sequences from SALK_015201. We further reconfirmed the deletion through a dCAPS assay as well as through Sanger sequencing of the *PHYB* genomic locus (Figure 4 and Figure S4). The missing “T” nucleotide at position 3,370 in the *PHYB* cDNA, converts a leucine codon to a premature stop codon that likely results in a truncation of 48 amino acids from its C‐terminal domain (Figure 4a). Other C‐terminal module disruptions to the PHYB protein have been reported to disrupt PHYB activity through various mechanisms (Qiu et al., 2019; Rockwell et al., 2006; Wagner & Quail, 1995). Previous studies have shown that the dimerization of the PHYB C‐terminal domain is essential for its accumulation in subnuclear photobodies as well as its interaction with PIFs driving their photoactivated degradation (Chen et al., 2003; Qiu et al., 2019). The catalytic ATP‐binding domain of PHYB contains four conserved subdomains named N, G1, F, and G2 that are essential for its histidine kinase‐related domain‐mediated dimerization (Qiu et al., 2019; Schneider‐Poetsch et al., 1991; Yeh & Lagarias, 1998). The *slim shady* allele of *PHYB* we have discovered contains an indel that would result in a truncation of the entire G2 subdomain, which would likely generate a loss of function allele. The loss of *PHYB* function is further evident from the long hypocotyl phenotype of *slim shady* that is indistinguishable from *phyB‐9* (Figure 1), another well‐validated knock‐out allele of *PHYB* (Reed et al., 1994).

Light‐activated phytochromes phosphorylate the PIF proteins and target them for 26S proteasomal‐mediated degradation. PIFs transcriptionally regulate expression of downstream growth factors and hence pave the mechanism by which phytochromes post‐translationally regulate photomorphogenesis (Al‐Sady et al., 2006; Hornitschek et al., 2012; Park et al., 2012). Our transcriptomic data show that a subset of` photomorphogenic suppressors such as *BBX28*, *PIF4*, *PIF3*, *PIL1*, and *PIL6* are upregulated in *slim shady* as compared to Col‐0 that are otherwise tightly regulated. Hence, in addition to degradation of PIFs, photo‐activated PHYB might be promoting photomorphogenesis through another mechanism in which they suppress the expression of these photomorphogenic suppressors. In future, this multilevel (transcriptional and post‐translational) regulation of PIF transcription factors by PHYB and possibly other phytochromes can be genetically and biochemically explored with the help of non‐phosphorylatable *PIF* transgenic lines.

Transcription factors belonging to BZR, ARF, and PIF families, otherwise known as the BAP module, work synergistically to promote the transcription of genes such as *IAA19*, *SAUR15*, and *PRE1* that are involved in positively regulating hypocotyl growth (Bouré et al., 2019). Our transcriptomic data show that these downstream growth regulators are collectively elevated in the *slim shady* allele, which can explain the elongated hypocotyl phenotype. In addition, our observation fits with the available knowledge that PHYB suppresses the expression *SAUR15* and *PRE1* by directly interacting with their respective transcriptional activators BES1 and BZR1 and inhibits their promoter binding (Wu et al., 2019). PIF4 competitively inhibits PHYB‐BZR1 interaction, therefore possibly attenuating the inhibitory effect of PHYB on the transcriptional activity of BZR1 (Dong et al., 2020). Because these targets are also elevated in *slim shady*, we predict that altered PHYB activity is responsible for the observed altered gene expression patterns.

The uninhibited transcriptional activity of PIFs results in an overaccumulation of auxin followed by a strong auxin response (Ren & Gray, 2015). Auxin induces apoplast acidification through activation of H^+^‐ATPase proton pumps, leading to a cascade of multiple events that in concert contribute towards cell wall expansion, including activation of potassium ion channels and stimulating carbohydrate remodeling proteins such as EXPs and XTHs (Majda & Robert, 2018). Influx of potassium into the cytosol triggers water uptake that builds tensile stress on the cell wall. Simultaneously, proteins like EXPs and XTHs induce cell wall loosening by disintegrating polysaccharide and glycosidic linkages that support the foundation of plant cell wall. In the case of *slim shady*, the overexpression of numerous *EXPs* (*EXPA3*, *EXPA8*, *EXPA11*, *EXLA1*, and *EXLA2*) and *XTHs* (*XTH4*, *XTH15*, *XTH17*, *XTH19*, *XTH24*, and *XTH30*) are consistent with the observed elongated hypocotyl phenotype.

*SAURs* are a class of early auxin responsive genes that facilitate hypocotyl growth by inducing cell expansion. They are triggered by upstream signals from either of the auxin, BR, or light‐signaling pathways and activate the plasma membrane H^+^‐ATPases by directly binding with and suppressing their inhibitors PP2C‐D subfamily of type 2C protein phosphatases (Ren et al., 2018; Spartz et al., 2014; Takahashi et al., 2012). Specifically, over expression of *SAUR15* manifests elongated hypocotyl by facilitating auxin biosynthesis and cell wall expansion via. activation of H^+^‐ATPases in *Arabidopsis* (Spartz et al., 2014). Transcriptomic data from the novel *PHYB* loss of function allele *slim shady* hint towards a molecular framework for light activated auxin‐mediated control of plant cell wall expansion where the loss of PHYB mediated transcriptional inhibition leads to overexpression of genes like *SAUR15*, *SAUR19*, *SAUR21*, and *SAUR36* that have been implicated for promoting hypocotyl growth (Spartz et al., 2012, 2014; Stamm & Kumar, 2013).

Previous studies have implicated negative photoregulation of *PHYA* expression mediated by PHYB, where *PHYA* gets highly expressed in a *phyB* loss of function background (Cantón & Quail, 1999). As anticipated, the transcript levels of *PHYA* were found to be elevated in *slim shady* as compared to WT. Additionally, PHYA is known to promote hypocotyl growth in light grown *Arabidopsis* seedlings, caused by the reduction/absence of biologically active phyB‐Pfr (Casal, 1996). Overexpression of PHYA alone has been attributed with the inhibition of hypocotyl growth (Boylan & Quail, 1991). Interestingly, another major UVA/Blue light‐sensing photoreceptor *PHOT1* showed increased expression in *slim shady*, indicating that PHYB may play a role in *PHOT1* expression in a similar fashion to *PHYA* (Sullivan et al., 2016).

In response to auxin, very few genes are either induced or repressed in SALK_015201 compared to WT. We found 91 genes that are upregulated and 21 genes that are downregulated in both Col‐0 and SALK_015201 (Figure S8 and Table S1). Notably, significantly fewer genes were induced by auxin in SALK_015201 compared to WT. Such an altered auxin response in this mutant line could be a consequence of loss of *PHYB*, a loss of *IRR* and/or the loss of both genes. In general, the lack of an observed robust auxin response in SALK_015201 at the molecular level fits with the elongated hypocotyl phenotype.

Collectively our initial phenotypic and molecular characterization of SALK_015201 suggests that the new *phyB* allele, *slim shady*, found within this T‐DNA background may be useful for the photoreceptor and/or auxin community. This study also has implications for functional genomic studies involving T‐DNA lines and serves as a reminder that careful genetic analyses require characterization more than one allele and/or complementation via transgenic approaches to provide conclusive evidence when examining gene function (Bergelson et al., 2016). We encourage all Arabidopsis researchers working with T‐DNA lines to confirm target gene expression, obtain more than one T‐DNA allele, generate additional alleles via targeted gene editing, or complement with a transgene or BAC when possible, in order to confidently ascribe gene function. Original T‐DNA collections, many of which were phenotypically screened by numerous laboratories, may contain a wealth of untagged background mutations which could now be identified using next generation sequencing.

## CONFLICT OF INTEREST

The authors have no conflict of interest to disclose.

## AUTHOR CONTRIBUTIONS

L.D., D.R.K., and B.P. D. designed the research; L.D., L.M., R.E.M., C.M., D.R.K., and J.W.W. performed the research; L.D., R.E.M, C.M., B.P.D., and D.R.K analyzed the data; L.D. and D.R.K with input from all authors wrote the paper.

## Supporting information

Fig S1‐S8

Table S1

Table S2

Table S3
