# Supplementary material for: slim shady is a novel allele of PHYTOCHROME B present in the T‐DNA line SALK_015201
Source: Plant Direct. 2021 Jun 12;5(6):e00326. doi: 10.1002/pld3.326 (PMC8197431; doi:10.1002/pld3.326)

Supplemental Figure 1: qPCR of key marker genes.

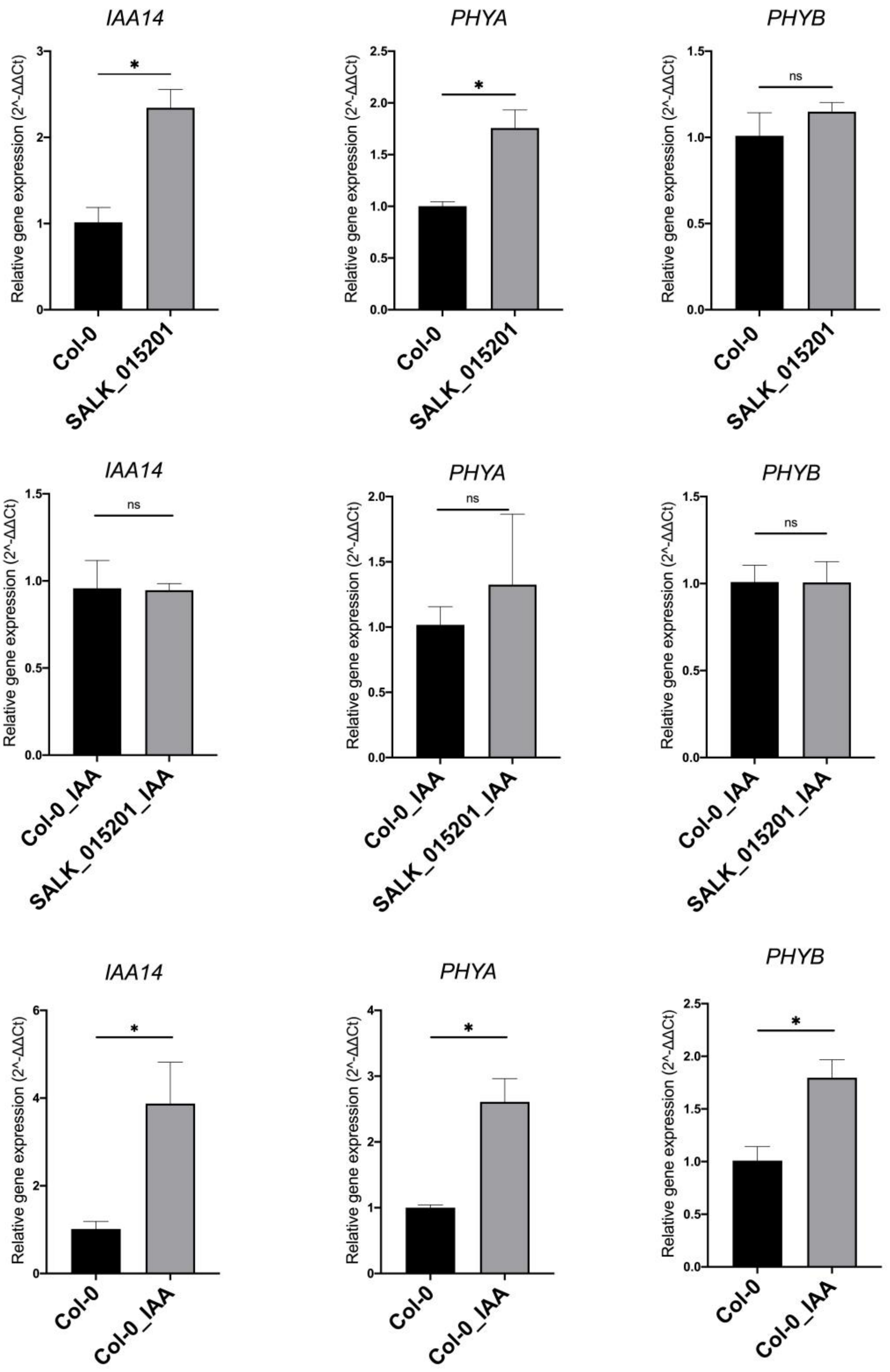

Supplemental Figure 2: GO network for up-regulated genes in SALK_015201 as compared to Col-0.

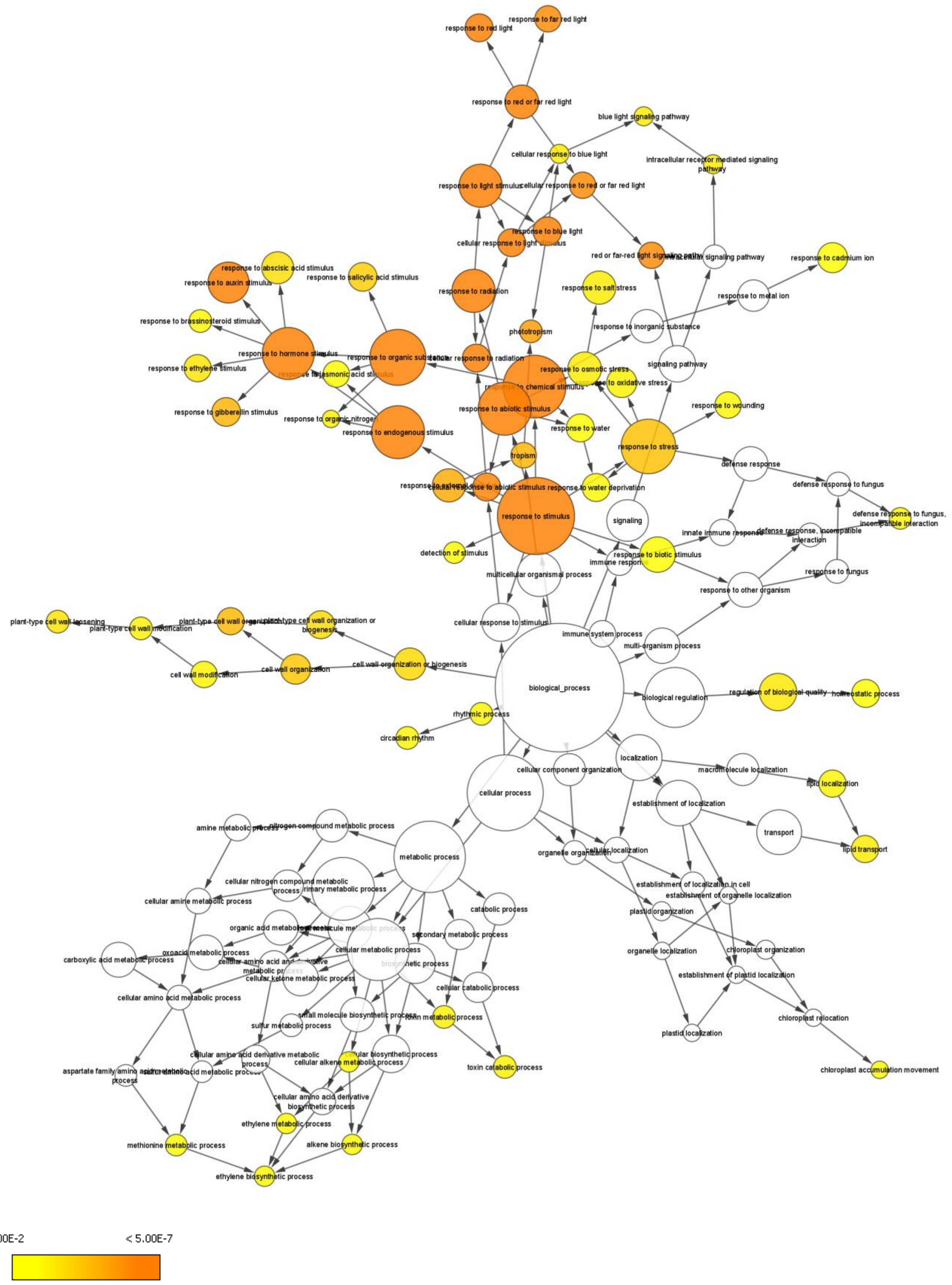

Supplemental Figure 3: GO network for down-regulated genes in SALK_015201 as compared to Col-0.

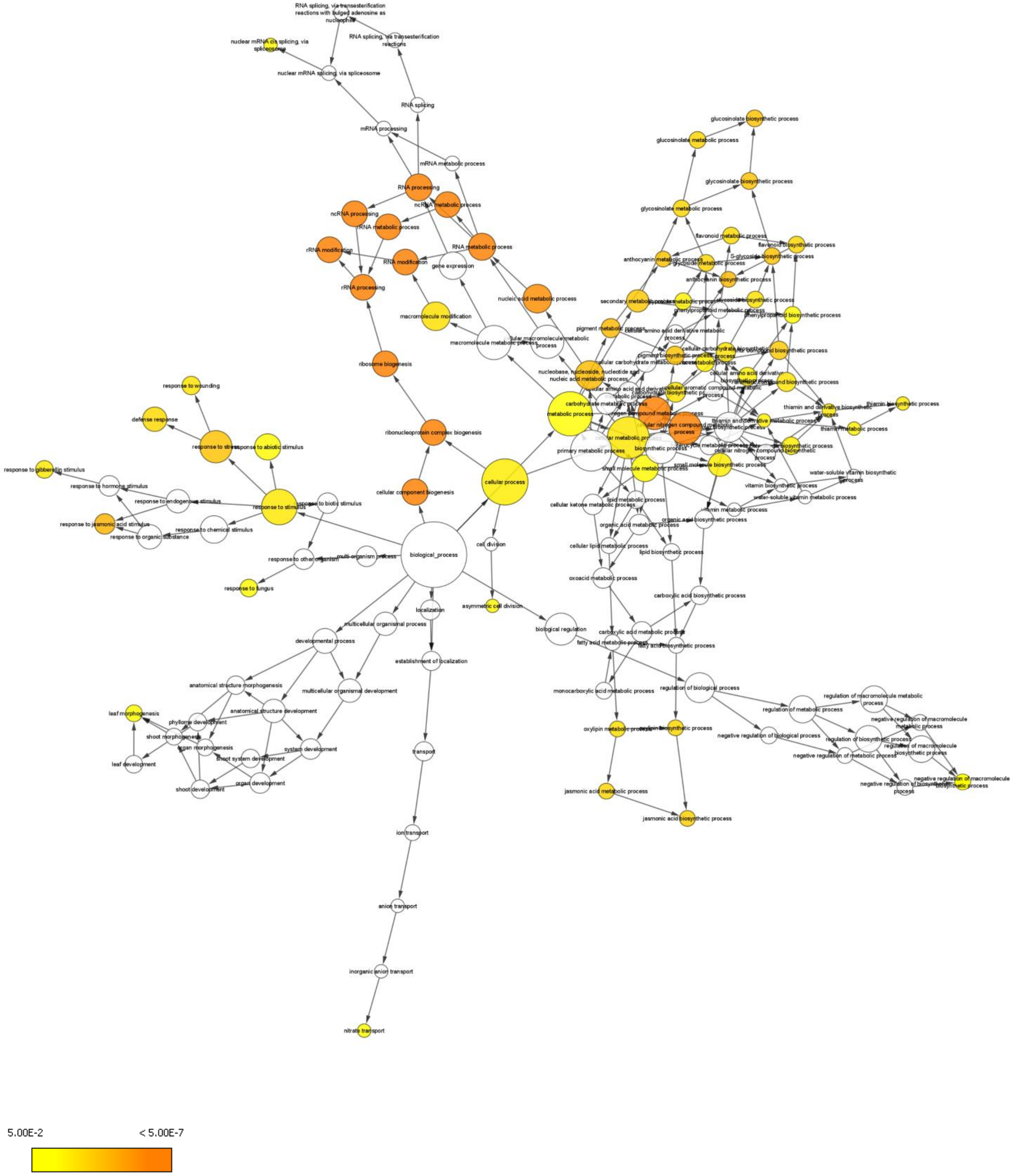

Supplemental Figure 4: GO network for up-regulated genes in SALK_015201 treated with 1 µM IAA for 24 hours as compared to Col-0 treated with 1 µM IAA for 24 hours.

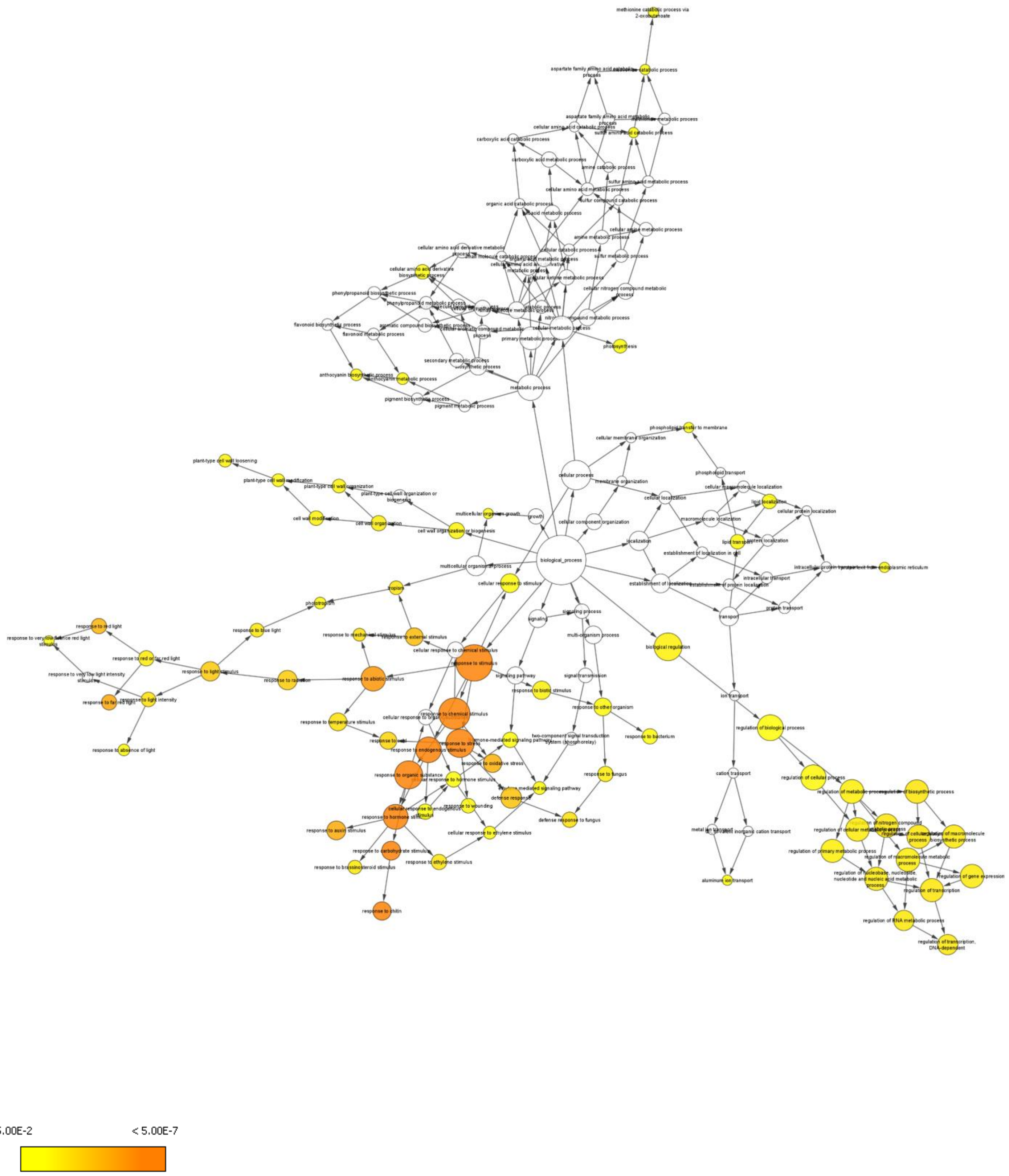

Supplemental Figure 5: GO network for down-regulated genes in SALK_015201 treated with 1 µM IAA for 24 hours as compared to Col-0 treated with 1 µM IAA for 24 hours.

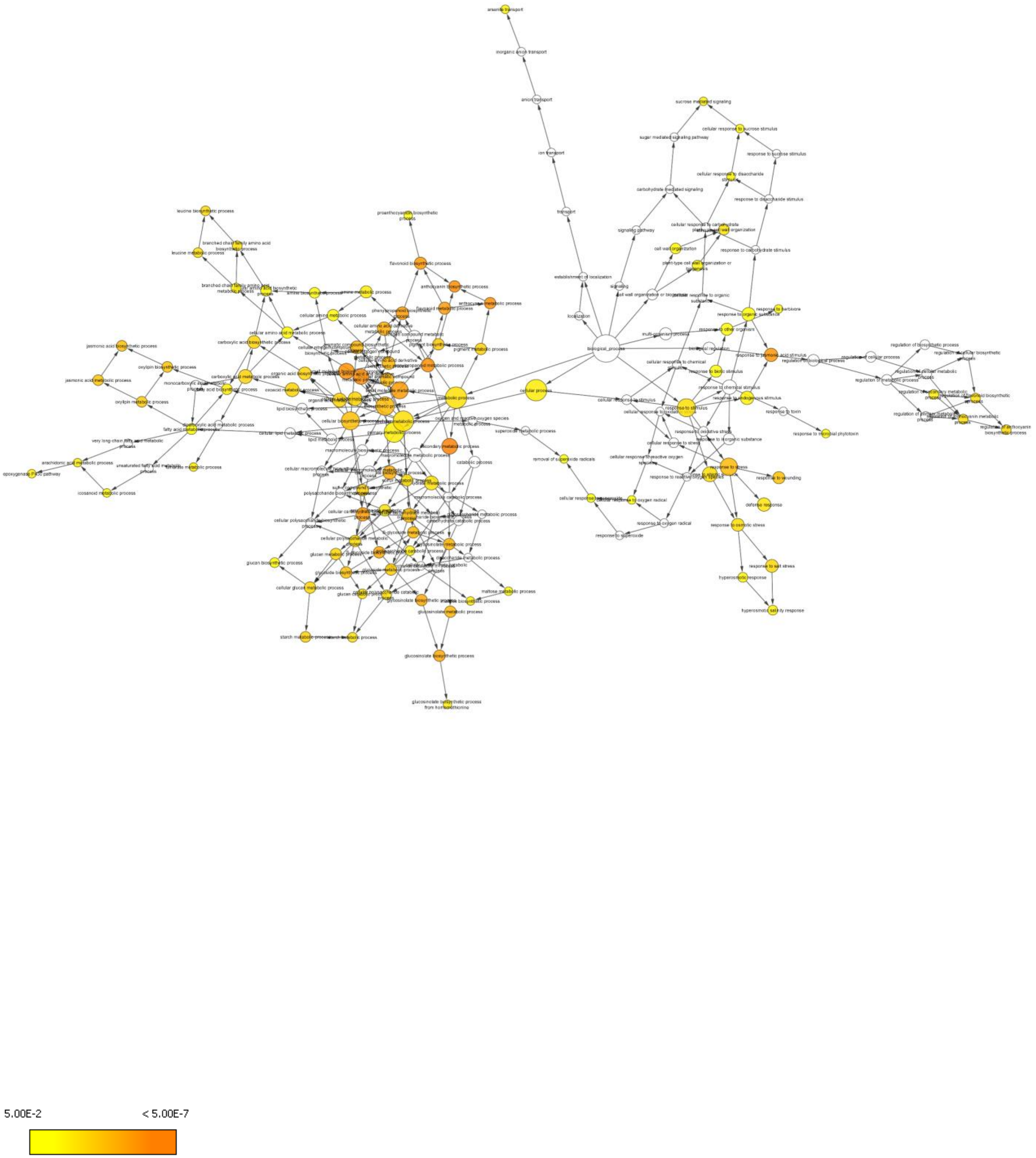

Supplemental Figure 6: P-value distribution of differentially expressed genes from our 3’ RNA seq data set.

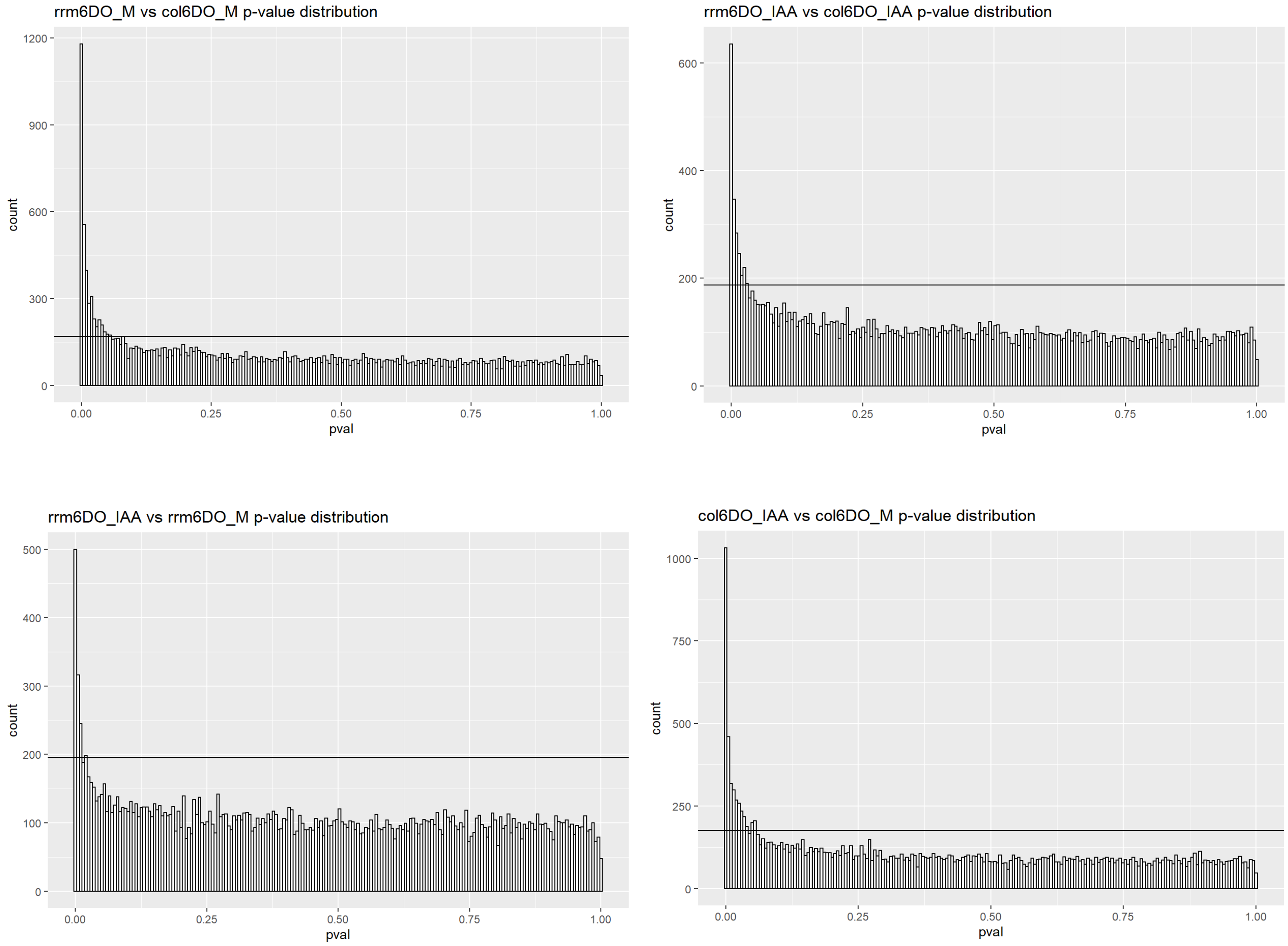

Supplemental Figure 7: Multiple sequence alignment showing the site of single nucleotide deletion in the *PHYB* locus of SALK_015201 with Col-0 and SALK_066572 as controls and PHYB genomic DNA as reference.

```
SALK_015201_phyB_F     ------------------------------------------------------------   0
Col-0_phyB_F           ------------------------------------------------------------   0
SALK_066572_phyB_F     ------------------------------------------------------------   0
phyB_genomic_DNA       TTATTTTGTACATAAAGAAAATGAATTTGGTTTGGTTAATTACGAATTTGATTTAGGCGT   3720
SALK_066572_phyB_R     ----------------------------------------------------TTGGGGTT   8
SALK_015201_phyB_R     ---------------------------------------------------TTTGGGGTT   9
Col-0_phyB_R           --------------------------------------------------ATTTTGGGGT   10

SALK_015201_phyB_F     -----------------------ACCATTCCATTTGTTTTGGTTATTGTTTAGTTGGAAC   37
Col-0_phyB_F           -----------------------CCCATTCTATTTGTTTTGGTTATTGTTTAGTTGGAAC   37
SALK_066572_phyB_F     -----------------------CCCATTCTATTTGTTTTGGTTATTGTTTAGTTGGAAC   37
phyB_genomic_DNA       TTAAAGAATTTGAGGTTTTAACCAATTCACTATTTGTTTTGGTTATTGTTTAGTTGGAAC   3780
SALK_066572_phyB_R     TAAAAAGATTTGAGGTTTTAACCAATTCACTATTTGTTTTGGTTATTGTTTAGTTGGAAC   68
SALK_015201_phyB_R     TTAAAAAATTTGAGGTTTTAACCAATTCACTATTTGTTTTGGTTATTGTTTAGTTGGAAC   69
Col-0_phyB_R           TTAAAAGATTTGAGGTTTTAACCAATTCACTATTTGTTTTGGTTATTGTTTAGTTGGAAC   70
                                                     * *****************************

SALK_015201_phyB_F     CTAGATTAGTTTGATTTTTGTATTCGGTTTAGTCGACTTGGGAACTTTTAGACACATCCA   97
Col-0_phyB_F           CTAGATTAGTTTGATTTTTGTATTCGGTTTAGTCGACTTGGGAACTTTTAGACACATCCA   97
SALK_066572_phyB_F     CTAGATTAGTTTGATTTTTGTATTCGGTTTAGTCGACTTGGGAACTTTTAGACACATCCA   97
phyB_genomic_DNA       CTAGATTAGTTTGATTTTTGTATTCGGTTTAGTCGACTTGGGAACTTTTAGACACATCCA   3840
SALK_066572_phyB_R     CTAGATTAGTTTGATTTTTGTATTCGGTTTAGTCGACTTGGGAACTTTTAGACACATCCA   128
SALK_015201_phyB_R     CTAGATTAGTTTGATTTTTGTATTCGGTTTAGTCGACTTGGGAACTTTTAGACACATCCA   129
Col-0_phyB_R           CTAGATTAGTTTGATTTTTGTATTCGGTTTAGTCGACTTGGGAACTTTTAGACACATCCA   130
                       ************************************************************

SALK_015201_phyB_F     TAGGCCTAGAATTAGCAGTCAAGGAATGTAATGTTTTCAAATTGATGAAAACCAGCTCAA   157
Col-0_phyB_F           TAGGCCTAGAATTAGCAGTCAAGGAATGTAATGTTTTCAAATTGATGAAAACCAGCTCAA   157
SALK_066572_phyB_F     TAGGCCTAGAATTAGCAGTCAAGGAATGTAATGTTTTCAAATTGATGAAAACCAGCTCAA   157
phyB_genomic_DNA       TAGGCCTAGAATTAGCAGTCAAGGAATGTAATGTTTTCAAATTGATGAAAACCAGCTCAA   3900
SALK_066572_phyB_R     TAGGCCTAGAATTAGCAGTCAAGGAATGTAATGTTTTCAAATTGATGAAAACCAGCTCAA   188
SALK_015201_phyB_R     TAGGCCTAGAATTAGCAGTCAAGGAATGTAATGTTTTCAAATTGATGAAAACCAGCTCAA   189
Col-0_phyB_R           TAGGCCTAGAATTAGCAGTCAAGGAATGTAATGTTTTCAAATTGATGAAAACCAGCTCAA   190
                       ************************************************************

SALK_015201_phyB_F     AAGTGTAAAACTTGGGTTTCATGTGTTGGTGTCTTTGTTATGTCTTTATTCGTTGTTTGC   217
Col-0_phyB_F           AAGTGTAAAACTTGGGTTTCATGTGTTGGTGTCTTTGTTATGTCTTTATTCGTTGTTTGC   217
SALK_066572_phyB_F     AAGTGTAAAACTTGGGTTTCATGTGTTGGTGTCTTTGTTATGTCTTTATTCGTTGTTTGC   217
phyB_genomic_DNA       AAGTGTAAAACTTGGGTTTCATGTGTTGGTGTCTTTGTTATGTCTTTATTCGTTGTTTGC   3960
SALK_066572_phyB_R     AAGTGTAAAACTTGGGTTTCATGTGTTGGTGTCTTTGTTATGTCTTTATTCGTTGTTTGC   248
SALK_015201_phyB_R     AAGTGTAAAACTTGGGTTTCATGTGTTGGTGTCTTTGTTATGTCTTTATTCGTTGTTTGC   249
Col-0_phyB_R           AAGTGTAAAACTTGGGTTTCATGTGTTGGTGTCTTTGTTATGTCTTTATTCGTTGTTTGC   250
                       ************************************************************

SALK_015201_phyB_F     AGAATGGCGTGTCCAGGTGAAGGTCTGCCTCCAGAGCTAGTCCGAGACATGTTCCATAGC   277
Col-0_phyB_F           AGAATGGCGTGTCCAGGTGAAGGTCTGCCTCCAGAGCTAGTCCGAGACATGTTCCATAGC   277
SALK_066572_phyB_F     AGAATGGCGTGTCCAGGTGAAGGTCTGCCTCCAGAGCTAGTCCGAGACATGTTCCATAGC   277
phyB_genomic_DNA       AGAATGGCGTGTCCAGGTGAAGGTCTGCCTCCAGAGCTAGTCCGAGACATGTTCCATAGC   4020
SALK_066572_phyB_R     AGAATGGCGTGTCCAGGTGAAGGTCTGCCTCCAGAGCTAGTCCGAGACATGTTCCATAGC   308
SALK_015201_phyB_R     AGAATGGCGTGTCCAGGTGAAGGTCTGCCTCCAGAGCTAGTCCGAGACATGTTCCATAGC   309
Col-0_phyB_R           AGAATGGCGTGTCCAGGTGAAGGTCTGCCTCCAGAGCTAGTCCGAGACATGTTCCATAGC   310
                       ************************************************************

SALK_015201_phyB_F     AGCAGGTGGACAAGCCCTGAAGGTTTAGGTCTAAGCGTATGTCGAAAGATT-TAAAGCTA   336
Col-0_phyB_F           AGCAGGTGGACAAGCCCTGAAGGTTTAGGTCTAAGCGTATGTCGAAAGATTTTAAAGCTA   337
SALK_066572_phyB_F     AGCAGGTGGACAAGCCCTGAAGGTTTAGGTCTAAGCGTATGTCGAAAGATTTTAAAGCTA   337
phyB_genomic_DNA       AGCAGGTGGACAAGCCCTGAAGGTTTAGGTCTAAGCGTATGTCGAAAGATTTTAAAGCTA   4080
SALK_066572_phyB_R     AGCAGGTGGACAAGCCCTGAAGGTTTAGGTCTAAGCGTATGTCGAAAGATTTTAAAGCTA   368
SALK_015201_phyB_R     AGCAGGTGGACAAGCCCTGAAGGTTTAGGTCTAAGCGTATGTCGAAAGATT-TAAAGCTA   368
Col-0_phyB_R           AGCAGGTGGACAAGCCCTGAAGGTTTAGGTCTAAGCGTATGTCGAAAGATTTTAAAGCTA   370
                       *************************************************** ********
```

```
SALK_015201_phyB_F      ATGAACGGTGAGGTTCAATACATCCGAGAATCAGAACGGTCCTATTTCCTCATCATTCTG      396
Col-0_phyB_F            ATGAACGGTGAGGTTCAATACATCCGAGAATCAGAACGGTCCTATTTCCTCATCATTCTG      397
SALK_066572_phyB_F      ATGAACGGTGAGGTTCAATACATCCGAGAATCAGAACGGTCCTATTTCCTCATCATTCTG      397
phyB_genomic_DNA        ATGAACGGTGAGGTTCAATACATCCGAGAATCAGAACGGTCCTATTTCCTCATCATTCTG      4140
SALK_066572_phyB_R      ATGAACGGTGAGGTTCAATACATCCGAGAATCAGAACGGTCCTATTTCCTCATCATTCTG      428
SALK_015201_phyB_R      ATGAACGGTGAGGTTCAATACATCCGAGAATCAGAACGGTCCTATTTCCTCATCATTCTG      428
Col-0_phyB_R            ATGAACGGTGAGGTTCAATACATCCGAGAATCAGAACGGTCCTATTTCCTCATCATTCTG      430
                        ************************************************************

SALK_015201_phyB_F      GAACTCCCTGTACCTCGAAAGCGACCATTGTCAACTGCTAGTGGAAGTGGTGACATGATG      456
Col-0_phyB_F            GAACTCCCTGTACCTCGAAAGCGACCATTGTCAACTGCTAGTGGAAGTGGTGACATGATG      457
SALK_066572_phyB_F      GAACTCCCTGTACCTCGAAAGCGACCATTGTCAACTGCTAGTGGAAGTGGTGACATGATG      457
phyB_genomic_DNA        GAACTCCCTGTACCTCGAAAGCGACCATTGTCAACTGCTAGTGGAAGTGGTGACATGATG      4200
SALK_066572_phyB_R      GAACTCCCTGTACCTCGAAAGCGACCATTGTCAACTGCTAGTGGAAGTGGTGACATGATG      488
SALK_015201_phyB_R      GAACTCCCTGTACCTCGAAAGCGACCATTGTCAACTGCTAGTGGAAGTGGTGACATGATG      488
Col-0_phyB_R            GAACTCCCTGTACCTCGAAAGCGACCATTGTCAACTGCTAGTGGAAGTGGTGACATGATG      490
                        ************************************************************

SALK_015201_phyB_F      CTGATGATGCCATATTAGTCACACTTCAGTTGGTATGAGAGTTTGTATCATTGTATGAGT      516
Col-0_phyB_F            CTGATGATGCCATATTAGTCACACTTCAGTTGGTATGAGAGTTTGTATCATTGTATGAGT      517
SALK_066572_phyB_F      CTGATGATGCCATATTAGTCACACTTCAGTTGGTATGAGAGTTTGTATCATTGTATGAGT      517
phyB_genomic_DNA        CTGATGATGCCATATTAGTCACACTTCAGTTGGTATGAGAGTTTGTATCATTGTATGAGT      4260
SALK_066572_phyB_R      CTGATGATGCCATATTAGTCACACTTCAGTTGGTATGAGAGTTTGTATCATTGTATGAGT      548
SALK_015201_phyB_R      CTGATGATGCCATATTAGTCACACTTCAGTTGGTATGAGAGTTTGTATCATTGTATGAGT      548
Col-0_phyB_R            CTGATGATGCCATATTAGTCACACTTCAGTTGGTATGAGAGTTTGTATCATTGTATGAGT      550
                        ************************************************************

SALK_015201_phyB_F      GTTTGTGTGTCTAACGACGTCGGAGGAGGATAGAAAGTTTTTTTTTTGTTTCCGGTGAGA      576
Col-0_phyB_F            GTTTGTGTGTCTAACGACGTCGGAGGAGGATAGAAAGTTTTTTTTTTGTTTCCGGTGAGA      577
SALK_066572_phyB_F      GTTTGTGTGTCTAACGACGTCGGAGGAGGATAGAAAGTTTTTTTTTTGTTTCCGGTGAGA      577
phyB_genomic_DNA        GTTTGTGTGTCTAACGACGTCGGAGGAGGATAGAAAGTTTTTTTTTTGTTTCCGGTGAGA      4320
SALK_066572_phyB_R      GTTTGTGTGTCTAACGACGTCGGAGGAGGATAGAAAGTTTTTTTTTTGTTTCCCGGGAGA      608
SALK_015201_phyB_R      GTTTGTGTGTCTAACGACGTCGGAGGAGGATAGAAAGTTTTTTTTTTTTTCCCGGGAGAT      608
Col-0_phyB_R            GTTTGTGTGTCTAACGACGTCGGAGGAGGATAGAAAGTTTTTTTTTTTTTCCCGGGAGAT      610
                        *********************************************** ** ** *

SALK_015201_phyB_F      TTAGTAGAGAAGAGGGAGATTATTTGCTTCCGCCNNNNNAA-------------------      617
Col-0_phyB_F            TTAGTAGAGAAGAGGGAGATTATTTGCTTCCGCCTTCAGCAA------------------      619
SALK_066572_phyB_F      TTAGTAGAGAAGAGGGAGATTATTTGCTTCCGGNNNCNNNAAA-----------------      620
phyB_genomic_DNA        TTAGTAGAGAAGAGGGAGATTATTTGCGTTCAGCTCAGCTCGCCGGAAAAAAAACGTAAC      4380
SALK_066572_phyB_R      TAGAGAAGGG--------------------------------------------------      618
SALK_015201_phyB_R      TGAGAAGGG---------------------------------------------------      617
Col-0_phyB_R            TGGGAAGGG---------------------------------------------------      619
                        *    *  *
```

Supplemental Figure 8: Overlap of auxin up- and down-regulated genes between Col-0 and SALK_015201C. (A) 21 genes are induced by IAA in both Col-0 and SALK_015201 (B) 91 genes are repressed by IAA in both Col-0 and SALK_015201.

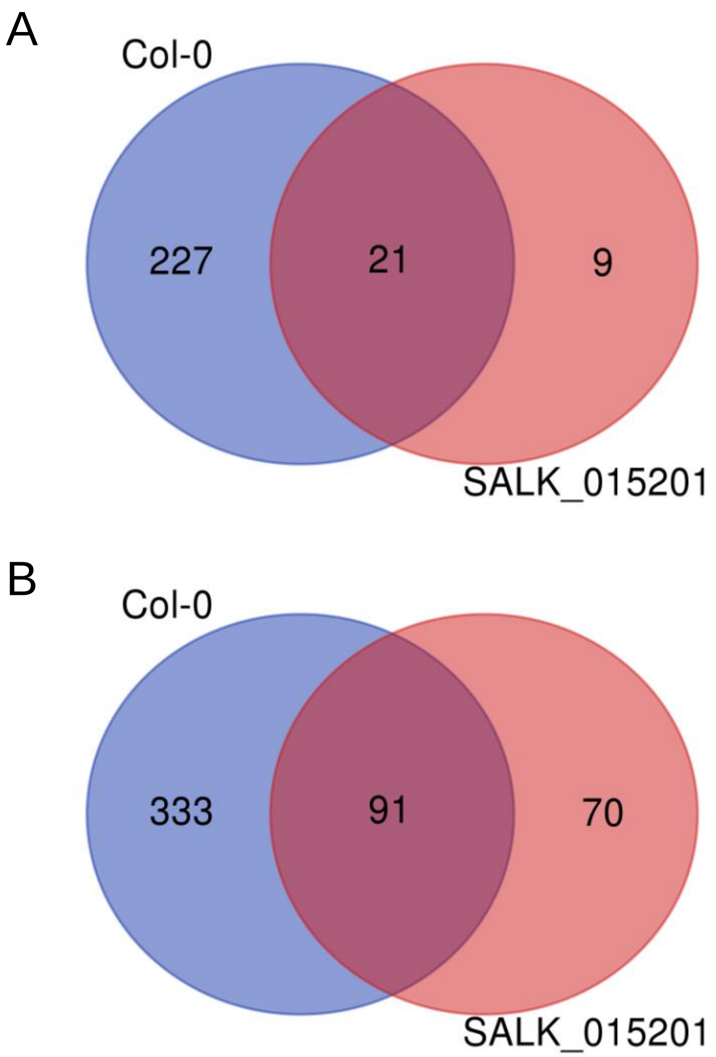

Supplement: Supplementary file 1 — Fig S1‐S8 [file PLD3-5-e00326-s004.pdf]
